# Over-Expression of Deubiquitinating Enzyme USP14 in Lung Adenocarcinoma Promotes Proliferation through the Accumulation of β-Catenin

**DOI:** 10.3390/ijms140610749

**Published:** 2013-05-23

**Authors:** Ning Wu, Cong Liu, Chong Bai, Yi-Ping Han, William C. S. Cho, Qiang Li

**Affiliations:** 1 Department of Respiratory Medicine, Changhai Hospital, Second Military Medical University, Shanghai 200433, China; E-Mails: wnrainbow@126.com (N.W.); congbai20122012@126.com (C.B.); yipinghan20122012@126.com (Y.-P.H.); 2 Department of Radiation Medicine, Second Military Medical University, Shanghai 200433, China; E-Mail: victorliu20102020@smmu.edu.cn; 3 Department of Clinical Oncology, Queen Elizabeth Hospital, Hong Kong, China; E-Mail: williamcscho@gmail.com

**Keywords:** USP14, NSCLC, prognosis, proliferation, β-catenin

## Abstract

The deubiquitinating enzyme USP14 has been identified and biochemically studied, but its role in lung cancer remains to be elucidated. The aim of this study was to evaluate the prognostic significance of USP14 in patients with lung adenocarcinoma and to define its role in lung cancer cell proliferation. USP14 mRNA levels in different non-small cell lung cancer (NSCLC) cell lines were detected by real-time qPCR. USP14 protein levels in surgically resected samples from NSCLC patients, and in NSCLC cell lines, were detected by immunohistochemistry or Western blot. The correlation of USP14 expression with clinical characteristics and prognosis was determined by survival analysis. After silencing USP14, cell proliferation was assessed by MTT assay and the cell cycle was measured by FACS assay. It was found that USP14 expression was upregulated in NSCLC cells, especially in adenocarcinoma cells. Over-expression of USP14 was associated with shorter overall survival of patients. Downregulation of USP14 expression arrested the cell cycle, which may be related to β-catenin degradation. Over-expression of USP14 was associated with poor prognosis in NSCLC patients and promoted tumor cell proliferation, which suggests that USP14 is a tumor-promoting factor and a promising therapeutic target for NSCLC.

## 1. Introduction

Lung cancer is one of the leading causes of cancer death in China and the USA [1–4]. It is also the leading cause of cancer-related mortality worldwide, with nearly 1.4 million deaths each year, and approximately 1.6 million new cases are diagnosed each year throughout the world [1]. The outcomes for patients with all stages of lung cancer have made some improvements in recent years. The use of systemic therapy in conjunction with local therapy has led to improved cure rates in both resectable and unresectable patient groups [1]. Non-small cell lung cancer (NSCLC), which includes adenocarcinoma, large cell carcinoma, bronchioloalveolar carcinoma, and squamous cell carcinoma, accounts for nearly 85% of all cases of lung cancer and is the most common pathological type of lung cancer [5,6]. The 5-year survival rate for NSCLC remains very poor worldwide [5]. Accordingly, it is important to obtain a better understanding of the molecular biology of lung cancer in order to develop more effective therapies.

Deubiquitinating enzymes (DUBs) function to remove covalently attached ubiquitin from proteins, thereby controlling substrate activity and/or abundance [7]. The USP family of DUBs plays an essential role in numerous cellular processes and signaling pathways [8–14]. Mammalian proteasomes are associated with three important DUBs: RPN11, UCH37, and USP14 [8,10]. USP14 is essential for life in eukaryotes and regulates many aspects of cell physiology. Studies have showed that USP14 can inhibit the proteasome *in vitro* and it can also inhibit protein turnover in cells [8]. In mice, defective USP14 results in ataxia and abnormal synaptic transmission [9] and USP14 is unique among those known USPs in that it is activated catalytically upon specific association with the 26S proteasome [11]. However, an understanding of the role of USP14 in cancer biology is still very limited. To date, only a few studies have implicated USP14 in cancer. Ishiwata, *et al.*, first found that the USP14 expression was upregulated in leukemic cells in 2001 [15], Shinji *et al.*, found that the USP14 expression in colorectal cancer is associated with liver and lymph node metastases in 2006, and Chuensumran, *et al.*, found that USP14 expression is associated with intrahepatic cholangiocarcinoma cell differentiation in 2011 [16,17]. However, to the best of our knowledge, there is as yet no report demonstrating a role for USP14 in lung adenocarcinoma.

Here we studied the expression and function of USP14 in NSCLC, its relationship with clinicopathological features, and its prognostic value for the survival of patients with lung adenocarcinoma. Finally, we investigated the possible role of USP14 in lung cancer proliferation and cell cycle regulation.

## 2. Results and Discussion

### 2.1. Expression of USP14 in Lung Adenocarcinoma Cell Lines

**Figure 1:**
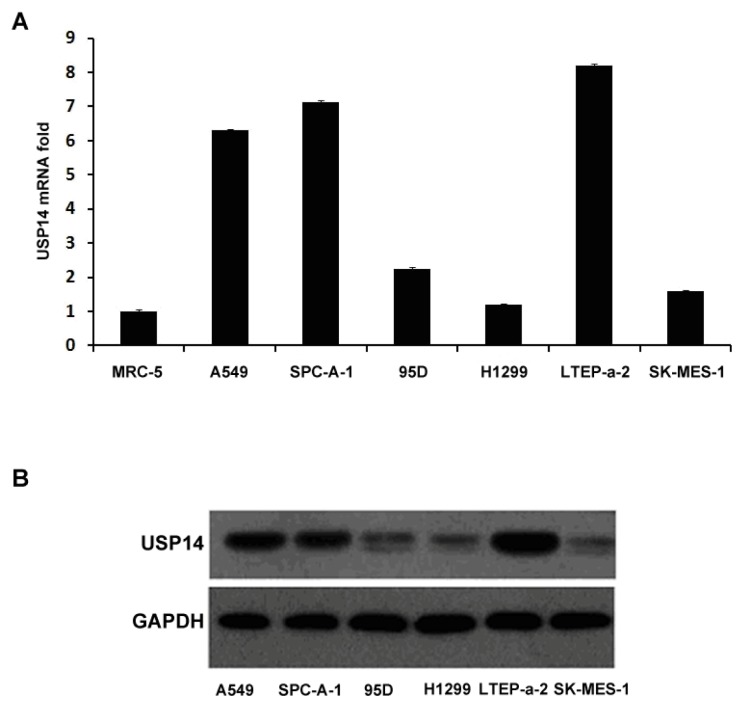
Expression of USP14 in lung adenocarcinoma cell lines. (**A**) Real-time qPCR analysis of USP14 mRNA expression in NSCLC cell lines using human pulmonary epithelial cell line (MRC-5) as normal control. The results were normalized for the amount of GAPDH serving as internal control; (**B**) Western blot analysis of USP14 protein expression in the NSCLC cell lines. GAPDH expression served as internal control.

Expression of USP14 mRNA was assayed in 7 human NSCLC cell lines and the normal control (human pulmonary epithelial cell line MRC-5). The level of USP14 mRNA was specifically upregulated in lung adenocarcinoma cell lines. Our data shows all the lung adenocarcinoma cells (A549, LTEP-a-2 and SPC-A-1) exhibited 6-fold or more increased USP14 mRNA as compared to the control, while the level of USP14 mRNA was unchanged in the other types of NSCLC cell lines (Figure 1A). To further validate the above results, the protein levels of USP14 in these cell lines were examined by Western blot assay; USP14 expression was consistently, upregulated in the lung adenocarcinoma cell lines (Figure 1B).

### 2.2. Expression of USP14 in NSCLC Tumor Tissues

**Figure 2:**
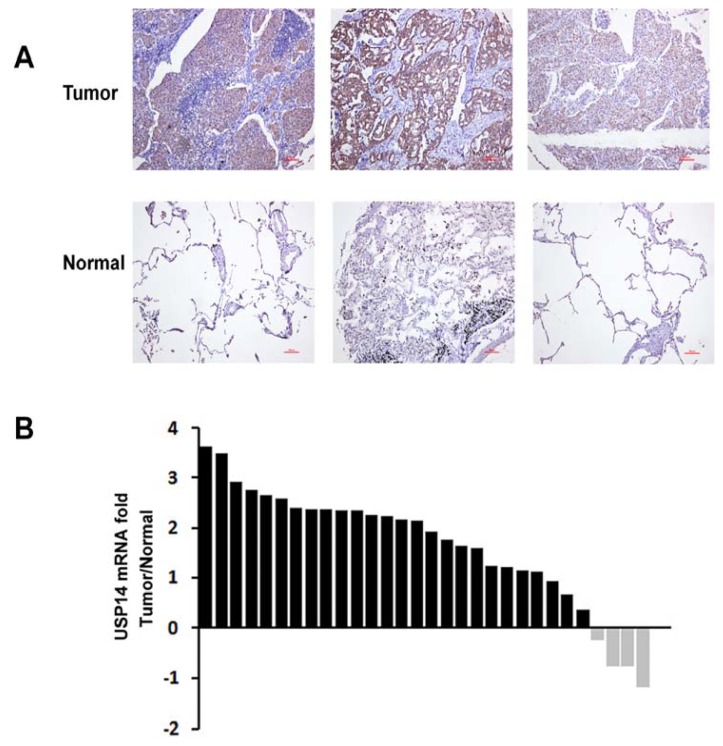
Expression of USP14 in NSCLC tumor tissues. (**A**) Representative images of USP14 immunohistochemistry staining in lung adenocarcinoma tissues and matched normal adjacent tissues. Red Bar, 100 μm; (**B**) Real-time qPCR analysis of USP14 mRNA expression in 30 lung adenocarcinoma tissues.

We first examined USP14 expression in 60 NSCLC tumor tissues and the matched tumor-adjacent normal tissues by immunohistochemistry (IHC) staining. High USP14 expression was detected in the malignant cells, while weak staining was observed throughout the matched normal control (Figure 2A). Specifically, USP14 expression was observed in 90.0% of lung adenocarcinoma specimens (27/30), 40.0% of the other types of NSCLC specimens (12/30), and 5.0% of the matched normal control specimens (3/60). High levels of USP14 expression (score: ++ ~ +++) were detected in the lung adenocarcinoma patients (17/30, 56.6%).

To further validate the IHC results, we also examined the mRNA levels of USP14 in 30 lung adenocarcinoma tissues and the matched tumor-adjacent normal tissues using real-time qPCR analysis. The result showed that USP14 was significantly increased in 26 of 30 adenocarcinoma tissues compared with the matched adjacent normal tissues (Figure 2B), suggesting that USP14 may be an important protein associated with the development of lung adenocarcinoma.

### 2.3. Correlation between USP14 Expression and Overall Survival in Lung Adenocarcinoma Patients

**Figure 3:**
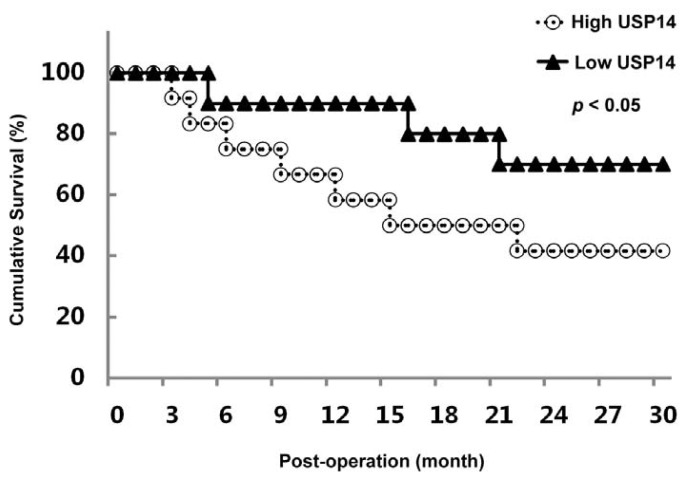
Kaplan-Meier plot of overall survival in lung adenocarcinoma patients post-operation according to the immunostaining results of USP14.

To evaluate the clinical significance of USP14 over-expression in lung adenocarcinoma, we investigated whether the levels of USP14 expression were associated with overall survival in lung adenocarcinoma. For the 30 lung adenocarcinoma patients, 22 had been followed-up for 3 years, and 3 cases were dropped. During the 3-year follow-up period, 10 out of 22 (45.5%) patients died as a result of disease progression. Kaplan-Meier curves indicated that patients with high USP14 expression (12 cases) had a significantly shorter overall survival (*p* < 0.05) than those with low USP14 expression (10 cases) (Figure 3).

### 2.4. Silencing USP14 Impaired Lung Adenocarcinoma Cell Proliferation Coupling with β-Catenin Reduction

**Figure 4:**
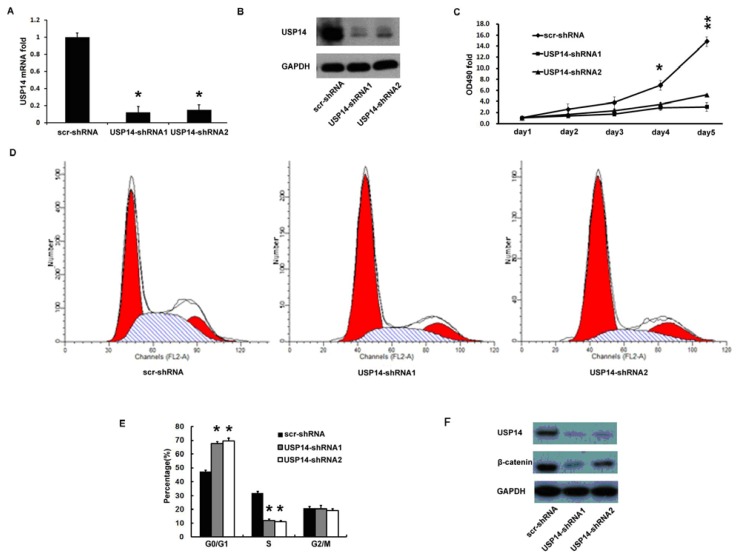
USP14 silencing impaired A549 cell proliferation coupling with β-catenin reduction. (**A**) Real-time qPCR analysis of the knockdown validity for USP14; (**B**) Western blot analysis of the knockdown validity for USP14; (**C**) MTT assay of A549 cell growth curves after transfection with USP14-shRNA lentivirus; (**D**) FACS assay of A549 cell cycle after transfection with USP14-shRNA lentivirus showed that: The cell number in S phase was decreased (in shadow), and the cell number in G0/G1 phase was significantly increased (in red); (**E**) Percent of cells in G0/G1, S and G2/M. Transfection increased the percent of G0/G1 phase cells, and decreased the percent of S phase cells. *p < 0.05; (**F**) Western blot analysis of correlation between USP14 and β-catenin. All the data was compared with the scramble control (*p < 0.05, **p < 0.01).

USP14 expression was higher in lung adenocarcinoma cell line A549, as shown in Figure 1. Therefore, two GFP labeled USP14 shRNA lentiviruses (USP14-shRNA1 and USP14-shRNA2) were transfected into the A549 cells, and the gene transfer efficiency evaluated by green fluorescence autograph was over 90% (Figure S1). The efficiency of USP14 silencing (nearly 80%) was assessed by real-time qPCR and Western blot assay (Figure 4A,B).

We detected the OD value of A549 cells by MTT assay to generate cell growth curves. The proliferation significantly decreased in the groups transfected with USP14 shRNA lentivirus at day 4 (*p* < 0.05) and day 5 (*p* < 0.01) (Figure 4C).

We further identified the role of USP14 in A549 cell cycle by FACS assay. The results demonstrated that the cell number in S phase was significantly decreased (*p* < 0.05) and the cell number in G0/G1 phase was significantly increased (*p* < 0.05) after transfection with USP14-shRNA lentivirus (Figure 4D).

To further explore the mechanism of the shift in A549 cell cycle profile after USP14 silencing, we used Western blot assay to investigate β-catenin, a key member in the Wnt pathway that promotes proliferation in different types of tumors [18–23], and is controlled through ubiquitination [24–26]. Interestingly, we found β-catenin protein levels were sharply decreased in conjunction with USP14 silencing in A549 cells (Figure 4E).

### 2.5. Discussion

To date, only a few studies have implicated USP14 in cancer [15–17], and to the best of our knowledge, this is perhaps the first report demonstrating the role of USP14 in lung adenocarcinoma. Previous studies showed that USP14 protein and mRNA levels were dysregulated in patients with more aggressive tumors of leukemia, colon cancer, and intrahepatic cholangiocarcinoma [15–17]. These papers are very important because they provide evidence that USP14 is implicated in cancer biology. In our study, we detected USP14 by IHC, and further validated this result by Western blot, and real-time qPCR assays. Our key finding has demonstrated that USP14 expression was specifically upregulated both in lung adenocarcinoma cell lines and tumor tissues, and was significantly correlated with overall survival of lung adenocarcinoma patients. Our current research investigated the role of USP14 in NSCLC, however further studies of USP14 on other tumor types may reveal that patients with higher levels of USP14 (regardless of mRNA or protein and/or regardless of primary tumor type) have poorer survival than patients with lower levels of USP14.

We further demonstrated that USP14 was important for cancer cell proliferation. Down-regulation of USP14 expression by lentivirus in NSCLC cell line led to decreased cell growth and cell cycle arrest, coupling with β-catenin degradation. These data suggested that USP14 might influence the biology of tumor cells. Further research will be needed to determine whether the over-expression of USP14 is an early or late event in lung tumorigenesis and the mechanism of over-expression. Another interesting question is why over-expression of USP14 is specific for adenocarcinoma. We have presented data suggesting that USP14 is upregulated in NSCLC and that it acts to increase cell growth and cell cycle. Thus, pharmacological suppression of USP14 may represent a promising approach for NSCLC treatment, especially for adenocarcinoma. Indeed, a selective small-molecule inhibitor of USP14 has been indentified in 2010 and 2012 [8,10].

The aberrant over-expression of USP14 in NSCLC may make it a good candidate as a therapeutic molecular target. Given that several studies have confirmed that USP14 can inhibit the proteasome *in vitro* [8,27,28] and can inhibit protein turnover in cells [8] the investigation and development of novel anticancer therapy based on inhibition of proteasome deubiquitinating activity or regulation of protein turnover is indicated. To date, some synthetic inhibitors of proteasome deubiquitinating have been made. In NSCLC, over-expression of USP14 appears to contribute to malignant progression, but the actual purpose of USP14 remains unknown. USP15, which is related to USP14, has been shown to play a role in SMAD signaling (29), and to stabilize the TGF-β receptor I and promote oncogenesis through the activation of TGF-β signaling in glioblastoma [29,30]. Whether USP14 has similar functions and whether it can regulate the TGF-β pathway remains unclear and we plan to investigate these issues in future studies.

## 3. Experimental Section

### 3.1. Patients

Surgical specimens from 60 NSCLC patients (these samples consisted of 30 squamous carcinomas and 30 adenocarcinomas) and matched normal control adjacent lung tissues were obtained postoperatively in 2008 from the Department of Respiratory Medicine, Changhai Hospital, Second Military Medical University (Shanghai, China). All patients gave signed, informed consent for their tissues to be used for scientific research. Ethical approval for the study was obtained from Changhai Hospital, Second Military Medical University (Shanghai, China). All diagnoses were based on pathological and/or cytological evidence. The histological features of the specimens were evaluated by senior pathologists according to the World Health Organization classification criteria. Tissues were obtained before chemotherapy and radiotherapy and were immediately frozen and stored at −80 °C prior to IHC and real-time qPCR assay. For the 30 adenocarcinoma patients, 22 had been followed-up for 3 years, including 10 deaths after operation (the other 8 were lost during the follow-up), and complete clinical data were electronically recorded.

### 3.2. Cell Culture

Primary human l pulmonary epithelial cell line MRC-5, and human NSCLC cell lines (A549, H1299, 95D, LTEP-a-2, SPC-A-1, and SK-MES-1) were obtained from the Cell Bank of Chinese Academy of Science (Shanghai, China) and cultured in DMEM medium (Hyclone, South Logan, UT, USA) supplemented with 10% fetal bovine serum (Hyclone), 2 mM l-glutamine and 100 μg/mL penicillin/streptomycin (Bio Light, Shanghai, China) as described in our previous studies [31].

### 3.3. Real-Time Quantitative Reverse Transcription Polymerase Chain Reaction

RNA was extracted with Trizol reagent (Invitrogen, Carlsbad, CA, USA) according to the manufacturer’s protocol. The cDNA synthesis and real-time qPCR was subsequently performed in triplicate using the Qiagen system as described detail in our previous studies [31–34]. Relative mRNA levels of USP14 were normalized to levels of the housekeeping gene GAPDH and calculated by the 2^−ΔΔCt^ method. The primers used are as follows: GAPDH (5′-CCATGTTCGTCATGGG-TGTGAACCA-3′ and 5′-GCCAGTAGAGGCAGGGATGATGTTG-3′) and USP14 (5′-GAGT-TGGACCTTT-CCAGA-3′ and 5′-TGCTTGCACAG-ATGTGA-3′).

### 3.4. Western Blot

Western blot analysis was performed according to a published method [33–35]. In brief, BCA Protein Assay Reagent Kit (Pierce, Rockford, IL, USA) was used to measure protein concentration. Equal amounts of protein were separated by 12% SDS-PAGE and transferred to nitrocellulose membranes (Schleicher and Schuell, Dassel, Germany). Blots were probed with antibodies specific for human USP14 (1:1000 dilution, Sigma-Aldrich, St. Louis, MO, USA) and human β-catenin (1:1000 dilution, Santa Cruz, CA, USA). Immunoblots were developed using with anti-rabbit-HRP conjugated secondary antibodies (Cell Signaling, Danvers, MA, USA). Supersignal West Femto Maximum Sensitivity substrate (Pierce) was used for the chemiluminescent visualization of proteins.

### 3.5. Immunohistochemistry

IHC staining was described previously [36]. Briefly, 4-μm thick sections were cut and anti-USP14 antibody (Sigma-Aldrich) was applied. Subsequent counterstaining was performed with hematoxylin. Immunostaining results for USP14 were evaluated using a semi-quantitative scoring system as described previously [37], which calculated the staining intensity and the percentage of positive cells. IHC staining was scored according to the following criteria: −, 0%–10% of the nucleated cells stained, +, 10%–40% stained, ++; 40%–70% stained and +++, 70%–100% stained. USP14 expression was considered to be observed when score ≥ +. Alternatively, IHC score of USP14 expression was (− ~ +) and (++ ~ +++), which represented low and high expression, respectively.

### 3.6. Cell Growth Assay

For cell growth assay, 500 cells per well were seeded in triplicate in a 96-well plate with complete growth medium. Cells were counted over 5 days using the MTT assay (Promega, Fitchburg, WI, USA) as described previously [34,38,39].

### 3.7. Cell Cycle Assay

After different treatments, cells were labeled with propidium iodide (PI) provided by BIPEC (Nanjing, Jiangsu, China), following the manufacturer’s instructions as described previously [33,40]. Samples were examined by FACS assay, and the results were analyzed using CellQuest software (Becton Dickinson, Franklin Lakes, NJ, USA) as described previously [34,38–40].

### 3.8. Lentivirus Vectors

Generation of USP14 specific RNAi or scramble control lentivirus vectors was performed by Shenggong Company (Shanghai, China). Cells were transfected with 1 mL of lentiviral supernatant containing equal dose (4 × 10^8^ PFU) for 2 h at a multiplicity of infection of 1:5, followed by incubation for 2 h at 37 °C as described previously [41–43]. The gene transfer efficiency was evaluated by GFP expression, which was detected by luminescence microscope (Leica, Berlin, Germany).

### 3.9. Statistical Analysis

Comparisons between experimental groups and relevant controls were performed by Student’s *t* test or ANOVA. Overall survival of patients was estimated by the Kaplan-Meier method, and the statistical significance of the differences was compared by the log-rank test. All the statistical analyses were performed with SPSS 16.0 (SPSS: Chicago, IL, USA, 2008) and *p* < 0.05 was considered statistically significant.

## 4. Conclusions

In summary, we found that USP14 was over-expressed in NSCLC patients. USP14 promotes NSCLC cell proliferation, which may be associated with β-catenin accumulation. The level of USP14 was significantly correlated with overall survival of lung adenocarcinoma patients. Further validation with functional analysis of the protein in the context of human carcinogenesis may assist in the development of novel therapeutic strategies for lung cancer.

## Supplementary Information



## References

[1] Ramalingam S.S., Owonikoko T.K., Khuri F.R. (2011). Lung cancer: New biological insights and recent therapeutic advances. CA Cancer J. Clin.

[2] Katlic M.R., Facktor M.A., Berry S.A., McKinley K.E., Bothe A., Steele G.D. (2011). Proven Care lung cancer: A multi-institutional improvement collaborative. CA Cancer J. Clin..

[3] Li Xinxin, Wang J., Xu Z., Ahmad A., Li E., Wang Y., Qin S., Wang Q. (2012). Expression of Sox2 and Oct4 and their clinical significance in human non-small-cell lung cancer. Int. J. Mol. Sci..

[4] Wen C., Dehnel T. (2011). China wrestles with lung cancer. Lancet Oncol.

[5] Wakelee H.A., Chang E.T., Gomez S.L., Keegan T.H., Feskanich D., Clarke C.A., Holmberg L., Yong L.C., Kolonel L.N., Gould M.K., West D.W. (2007). Lung cancer incidence in never smokers. J. Clin. Oncol.

[6] Fujii T., Dracheva T., Player A., Chacko S., Clifford R., Strausberg R.L., Buetow K., Azumi N., Travis W.D., Jen1 J. (2002). A preliminary transcriptome map of non-small cell lung cancer. Cancer Res.

[7] Sowa M.E., Bennett E.J., Gygi S.P., Harper J.W. (2009). Defining the human deubiquitinating enzyme interaction landscape. Cell.

[8] Lee B.H., Lee M.J., Park S., Oh D.-C., Elsasser S., Chen P.-C., Gartner C., Dimova N., Hanna J., Gygi S.P. (2010). Enhancement of proteasome activity by a small-molecule inhibitor of USP14. Nature.

[9] Anderson C., Crimmins S., Wilson J.A., Korbel G.A., Ploegh H.L., Wilson S.M. (2005). Loss of Usp14 results in reduced levels of ubiquitin in ataxia mice. J. Neurochem.

[10] Nag D.K., Finley D. (2012). A small-molecule inhibitor of deubiquitinating enzyme USP14 inhibits Dengue virus replication. Virus Res.

[11] Peth A., Besche H.C., Goldberg A.L. (2009). Ubiquitinated proteins activate the proteasome by binding to Usp14/Ubp6, which causes 20S gate opening. Mol. Cell.

[12] Mines M.A., Goodwin J.S., Limbird L.E., Cui F.F., Fan G.H. (2009). Deubiquitination of CXCR4 by USP14 is critical for both CXCL12-induced CXCR4 degradation and chemotaxis but not ERK ativation. J. Biol. Chem.

[13] Wilson S.M., Bhattacharyya B., Rachel R.A., Coppola V., Tessarollo L., Householder D.B., Fletcher C.F., Miller R.J., Copeland N.J., Jenkins N.A. (2002). Synaptic defects in ataxia mice result from a mutation in Usp14, encoding a ubiquitin-specific protease. Nat. Genet.

[14] Borodovsky A., Kessler B.M., Casagrande R., Overkleeft H.S., Wilkinson K.D., Ploegh H.L. (2001). A novel active site-directed probe specific for deubiquitylating enzymes reveals proteasome association of USP14. EMBO J.

[15] Ishiwata S., Katayama J., Shindo H., Ozawa Y., Itoh K., Mizugaki M. (2001). Increased expression of queuosine synthesizing enzyme, tRNA-guanine transglycosylase, and queuosine levels in tRNA of leukemic cells. J. Biochem.

[16] Shinji S., Naito Z., Ishiwata S., Ishiwata T., Tanaka N., Furukawa K., Suzuki H., Seya T., Matsuda A., Katsuta M., Tajiri T. (2006). Ubiquitin-specific protease 14 expression in colorectal cancer is associated with liver and lymph node metastases. Oncol. Rep.

[17] Chuensumran U., Saelee P., Punyarit P., Wongkham S., Pairojkul C., Chauin S., Petmitr S. (2011). Ubiquitin-specific protease 14 expression associated with intrahepatic cholangiocarcinoma cell differentiation. Asian Pac. J. Cancer Prev.

[18] Yang L., Chen Y., Cui T., Knösel1 T., Zhang Q., Albring K.F., Huber O., Petersen I. (2012). Desmoplakin acts as a tumor suppressor by inhibition of the Wnt/beta-catenin signaling pathway in human lung cancer. Carcinogenesis.

[19] Tsao C.M., Yan M.D., Shih Y.L., Yu P.N., Kuo C.C., Lin W.C., Li H.J., Lin Y.W. (2012). SOX1 functions as a tumor suppressor through antagonizing the WNT/beta-catenin signaling pathway in hepatocellular carcinoma. Hepatology.

[20] Subbaiah V.K., Narayan N., Massimi P., Banks L. (2012). Regulation of the DLG tumor suppressor by beta-catenin. Int. J. Cancer.

[21] Yoshioka S., King M.L., Ran S., Okuda H., MacLean J.A., McAsey M.E., Sugino N., Brard L., Watabe K., Hayashi K. (2012). WNT7A regulates tumor growth and progression in ovarian cancer through the WNT/beta-catenin pathway. Mol. Cancer Res.

[22] Shu X.S., Geng H., Li L., Ying J., Ma C., Wang Y., Poon F.F., Wang X., Ying Y., Yeo W. (2011). The epigenetic modifier PRDM5 functions as a tumor suppressor through modulating WNT/beta-catenin signaling and is frequently silenced in multiple tumors. PLoS One.

[23] Wang E.Y., Yeh S.H., Tsai T.F., Huang H.P., Jeng Y.M., Lin W.H., Chen W.C., Yeh K.H., Chen P.J., Chen D.S. (2011). Depletion of beta-catenin from mature hepatocytes of mice promotes expansion of hepatic progenitor cells and tumor development. Proc. Natl. Acad. Sci. USA.

[24] Sadot E., Simcha I., Iwai K., Ciechanover A., Geiger B., Ben-Ze’ev A. (2000). Differential interaction of plakoglobin and beta-catenin with the ubiquitin-proteasome system. Oncogene.

[25] Tran H., Polakis P. (2012). Reversible modification of adenomatous polyposis coli (APC) with K63-linked polyubiquitin regulates the assembly and activity of the beta-catenin destruction complex. J. Biol. Chem.

[26] Jeong W.J., Yoon J., Park J.C., Lee S.H., Lee S.H., Kaduwal S., Kim H., Yoon J.-B., Choi K.-Y. (2012). Ras stabilization through aberrant activation of Wnt/beta-catenin signaling promotes intestinal tumorigenesis. Sci. Signal..

[27] Chen P.C., Qin L.N., Li X.M., Walters B.J., Wilson J.A., Mei L., Wilson S.M. (2009). The proteasome-associated deubiquitinating enzyme Usp14 is essential for the maintenance of synaptic ubiquitin levels and the development of neuromuscular junctions. J. Neurosci.

[28] Hu M., Li P., Song L., Jeffrey P.D., Chernova T.A., Wilkinson K.D., Cohen R.E., Shi Y. (2005). Structure and mechanisms of the proteasome-associated deubiquitinating enzyme USP14. EMBO J.

[29] Inui M., Manfrin A., Mamidi A., Martello G., Morsut L., Soligo S., Enzo E., Moro S., Polo S., Dupont S. (2011). USP15 is a deubiquitylating enzyme for receptor-activated SMADs. Nat. Cell Biol.

[30] Eichhorn P.J., Rodon L., Gonzalez-Junca A., Dirac A., Gili M., Martínez-Sáez E., Aura C., Barba I., Peg V., Prat A. (2012). USP15 stabilizes TGF-beta receptor I and promotes oncogenesis through the activation of TGF-beta signaling in glioblastoma. Nat. Med.

[31] Wu N., Gu H.J., Li Q. (2010). Effects of antidiabetic drug metformin on the migration and invasion abilities of human pulmonary adenocarcinoma A549 cell line *in vitro*. J. Thorac. Dis.

[32] Hao J., Zhang S., Zhou Y., Hu X., Shao C. (2011). MicroRNA 483-3p suppresses the expression of DPC4/Smad4 in pancreatic cancer. FEBS Lett.

[33] Hao J., Zhang S., Zhou Y., Liu C., Hu X., Shao C. (2011). MicroRNA 421 suppresses DPC4/Smad4 in pancreatic cancer. Biochem. Biophys. Res. Commun.

[34] Liu C., Li B., Cheng Y., Lin J., Hao J., Zhang S., Mitchel R.E.J., Sun D., Ni J., Zhao L. (2011). MiR-21 plays an important role in radiation induced carcinogenesis in BALB/c mice by directly targeting the tumor suppressor gene Big-h3. Int. J. Biol. Sci.

[35] Chen T., Guo J., Yang M., Han C., Zhang M., Chen W., Liu Q., Wang J., Cao X. (2004). Cyclosporin A impairs dendritic cell migration by regulating chemokine receptor expression and inhibiting cyclooxygenase-2 expression. Blood.

[36] Xu X.S., Wang L., Abrams J., Wang G. (2011). Histone deacetylases (HDACs) in XPC gene silencing and bladder cancer. J. Hematol. Oncol.

[37] Lv T., Yuan D., Miao X., Lv Y., Zhan P., Shen X., Song Y. (2012). Over-expression of LSD1 promotes proliferation, migration and invasion in non-small cell lung cancer. PLoS One.

[38] Liu C., Gao F., Li B., Mitchel R.E.J., Liu X., Lin J., Zhao L., Cai J. (2011). TLR4 knockout protects mice from radiation-induced thymic lymphoma by downregulation of IL6 and miR-21. Leukemia.

[39] Liu C., Zhou C., Gao F., Cai S., Zhang C., Zhao L., Zhao F., Cao F., Lin J., Yang Y. (2011). MiR-34a in age and tissue related radio-sensitivity and serum miR-34a as a novel indicator of radiation injury. Int. J. Biol. Sci.

[40] Liu C., Lin J., Zhao L., Yang Y., Gao F., Li B., Cui J., Cai J. (2011). Gamma-ray irradiation impairs dendritic cell migration to CCL19 by down-regulation of CCR7 and induction of cell apoptosis. Int. J. Biol. Sci.

[41] Hofig I., Atkinson M.J., Mall S., Krackhardt A.M., Thirion C., Anastasov N. (2012). Poloxamer synperonic F108 improves cellular transduction with lentiviral vectors. J. Gene Med.

[42] Luche R.M., Enssle J., Kiem H.P. (2012). Abrogated cryptic activation of lentiviral transfer vectors. Sci. Rep.

[43] Wu D.T., Seita Y., Zhang X., Lu C.W., Roth M.J. (2012). Antibody-directed lentiviral gene transduction for live-cell monitoring and selection of human iPS and hES cells. PLoS One.

