# Supplementary material for: Over-Expression of Deubiquitinating Enzyme USP14 in Lung Adenocarcinoma Promotes Proliferation through the Accumulation of β-Catenin
Source: Int J Mol Sci. 2013 May 23;14(6):10749–60. doi: 10.3390/ijms140610749 (PMC3709700; doi:10.3390/ijms140610749)
Supplement: Supplementary file 1 [file ijms-14-10749-s001.pdf]

Supplementary Information

Figure S1. Green fluorescence autograph analysis of the gene transfer efficiency of the shRNA lentiviruses (USP14-shRNA1 and USP14-shRNA2) in A549 cells. Cells were transfected with 1 mL of lentiviral supernatant containing equal dose (4×10^8 pfu) for 2 h at a multiplicity of infection of 1:5, followed by incubation for 2 h at 37 °C and observed by luminescence microscope.

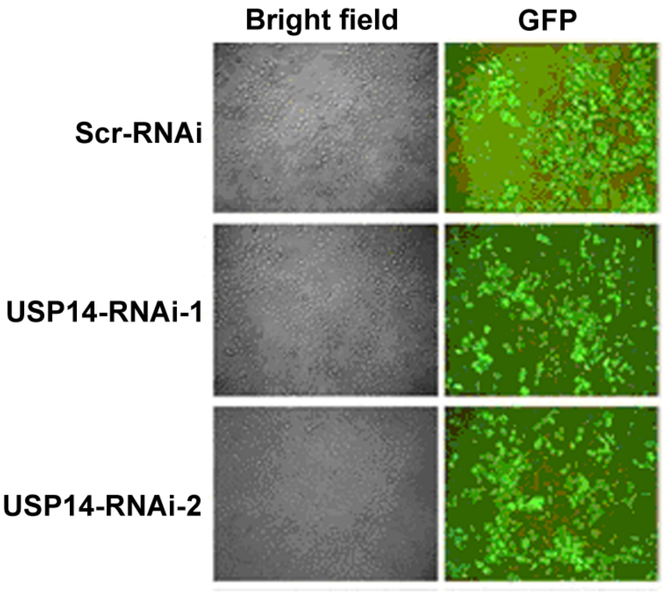

© 2013 by the authors; licensee MDPI, Basel, Switzerland. This article is an open access article distributed under the terms and conditions of the Creative Commons Attribution license (http://creativecommons.org/licenses/by/3.0/).
